# *Wolbachia* introduction into *Lutzomyia longipalpis* (Diptera: Psychodidae) cell lines and its effects on immune-related gene expression and interaction with *Leishmania infantum*

**DOI:** 10.1186/s13071-018-3227-4

**Published:** 2019-01-15

**Authors:** Daniela da Silva Gonçalves, Iñaki Iturbe-Ormaetxe, Andrea Martins-da-Silva, Erich Loza Telleria, Marcele Neves Rocha, Yara M. Traub-Csekö, Scott L. O’Neill, Maurício Roberto Viana Sant’Anna, Luciano Andrade Moreira

**Affiliations:** 1 Grupo Mosquitos Vetores: Endossimbiontes e Interação Patógeno Vetor, Centro de Pesquisas René Rachou - Fundação Oswaldo Cruz, Av. Augusto de Lima 1715, 30190-002. Belo Horizonte, Belo Horizonte, MG Brazil; 2 World Mosquito Program, Institute of Vector-Borne Disease, Monash University, 12 Innovation Walk, Clayton, VIC 3800 Australia; 3 Laboratório de Biologia Molecular de Parasitos e Vetores, Instituto Oswaldo Cruz - Fundação Oswaldo Cruz, Av. Brasil, 4365, Rio de Janeiro, RJ 21040-900 Brazil; 4 Laboratório de Insetos Hematófagos, Departamento de Parasitologia, Instituto de Ciências Biológicas/UFMG, Av. Antônio Carlos, 6627, 31270-901. Belo Horizonte, Belo Horizonte, MG Brazil

**Keywords:** *Wolbachia*, *Lutzomyia longipalpis*, Lulo cell, LL-5 cell, *Leishmania infantum*

## Abstract

**Background:**

The leishmaniases are important neglected diseases caused by *Leishmania* spp. which are transmitted by sand flies, *Lutzomyia longipalpis* being the main vector of visceral leishmaniasis in the Americas. The methodologies for leishmaniasis control are not efficient, causing 1.5 million reported cases annually worldwide, therefore showing the need for development of novel strategies and interventions to control transmission of the disease. The bacterium *Wolbachia pipientis* is being used to control viruses transmitted by mosquitoes, such as dengue and Zika, and its introduction in disease vectors has been effective against parasites such as *Plasmodium.* Here we show the first successful establishment of *Wolbachia* into two different embryonic cell lines from *L. longipalpis*, LL-5 and Lulo, and analysed its effects on the sand fly innate immune system, followed by *in vitro Leishmania infantum* interaction.

**Results:**

Our results show that LL-5 cells respond to *w*Mel and *w*MelPop-CLA strains within the first 72 h post-infection, through the expression of antimicrobial peptides and inducible nitric oxide synthase resulting in a decrease of *Wolbachia* detection in the early stages of infection. In subsequent passages, the *w*Mel strain was not able to infect any of the sand fly cell lines while the *w*MelPop-CLA strain was able to stably infect Lulo cells and LL-5 at lower levels. In *Wolbachia* stably infected cells, the expression of immune-related genes involved with downregulation of the IMD, Toll and Jak-Stat innate immune pathways was significantly decreased, in comparison with the uninfected control, suggesting immune activation upon *Wolbachia* transinfection. Furthermore, *Wolbachia* transinfection did not promote a negative effect on parasite load in those cells.

**Conclusions:**

Initial strong immune responses of LL5 cells might explain the inefficiency of stable infections in these cells while we found that Lulo cells are more permissive to infection with *Wolbachia* causing an effect on the cell immune system, but not against *in vitro L. infantum* interaction*.* This establishes Lulo cells as a good system for the adaptation of *Wolbachia* in *L. longipalpis*.

**Electronic supplementary material:**

The online version of this article (10.1186/s13071-018-3227-4) contains supplementary material, which is available to authorized users.

## Background

Leishmaniasis is a spectrum of important epidemiological diseases, endemic in 98 countries with over 1.5 million cases reported annually worldwide. About one billion people live in areas of high transmission risk [1, 2]. Visceral leishmaniasis (VL) is caused by *Leishmania donovani* in the Old World and *L. infantum* in parts of the Old World and New World [3], reaching up to 400,000 cases and around 40,000 deaths every year [2, 4, 5]. Currently, there is no vaccine for humans, so prevention and control of leishmaniasis are based on early diagnosis, effective drug administration [6] and protecting humans against the insect bite by using, for example, bednets, repellents and insecticide treatment [2, 7, 8].

Recently, the endosymbiont bacterium *Wolbachia* has been used as an alternative strategy to control vector-borne diseases, through the reduction or blocking of pathogen infections. This bacterium naturally infects around 40 to 70% of arthropods and some nematodes, being maternally transmitted through the eggs to subsequent generations [9–11]. The broad natural prevalence of *Wolbachia* in invertebrates has prompted studies on its potential to protect the host against pathogens. Previous studies have shown that the presence of *Wolbachia* can protect *Drosophila* against RNA viruses [12, 13] and its presence can induce the upregulation of immune genes, such as Relish and Dorsal, and also antimicrobial peptides (AMPs), i.e. attacin and diptericin [14]. Later, different strains of this bacterium were introduced into mosquitoes, upregulating immune related genes, such as TEP1, Myd88, SOCS36E, Cactus and the AMPs Defensin and Cepropin. This led to the reduction of infection by pathogens that cause different diseases such as dengue, chikungunya, malaria and Zika [15–22].

*Wolbachia* has been detected in sand flies of the genera *Phlebotomus* and *Lutzomyia*, but the impact of *Wolbachia* on the *Leishmania* infection load has not been reported*. Phlebotomus papatasi* and *Phlebotomus perniciosus* are naturally infected with strains *w*Pap and *w*Prn, respectively, whereas both *Lutzomyia shanonni* and *Lutzomyia whitmani* are infected with the strain *w*Whi [11, 23, 24]. *Lutzomyia longipalpis*, the main vector of *L. infantum* in the Americas [25, 26], was not found to be naturally infected with *Wolbachia* in some studies [11, 23, 26]. However, more recently, *Wolbachia* was detected with a low infection rate in *L. longipalpis* in a small population in Brazil, which suggests either a rare event of horizontal transmission by the feeding habits of larvae, with the possible acquisition of *Wolbachia* from decomposing bodies of arthropods, or a localised infection, considering that *L. longipalpis* is a species complex [27].

In order to successfully transinfect *Wolbachia* into a new host, previous studies have suggested culturing *Wolbachia* from the original host in cell lines belonging to the target species, in order to facilitate the bacteria adaptation to this new organism [21, 28]. After numerous unsuccessful attempts, the *Wolbachia* strain *w*MelPop-CLA from *Drosophila melanogaster* was introduced into *Aedes aegypti* mosquito embryos through microinjections, following its adaptation to *Aedes* cell lines for several months. *Wolbachia* was able to be established and spread into numerous tissues of the adult mosquitoes, to be vertically transmited to their offspring and to transfer some of its *Drosophila* phenotypes (reduction in longevity and cytoplasmic incompatibility) into the mosquito host [29, 30]. Furthermore, the same *Wolbachia* strain caused the upregulation of a range of immune-related genes, such as TEPs, prophenoloxidase and AMPs, whereas some genes from the Toll and IMD pathways were downregulated [31, 32].

Most of the studies involving *Wolbachia* are focused on transinfection into mosquitoes and the effects of the infection on the new host. Considering the importance of leishmaniasis on human health, it is crucial to investigate novel control strategies, mainly because sand fly control through insecticides may be hindered by insecticide resistance [33]. Other potential drawbacks for successful insect control include vector urbanisation [34] and difficulties finding immature stages in nature [35]. Here we tested the possibility of *Wolbachia* infection into *L. longipalpis* sand fly cell lines as a first step towards using this bacterium to control leishmaniasis.

In our experiments, we used two embryonic *L. longipalpis* cell lines: the LL-5 cell line, which consists of at least two cell types, epithelioid and fibroblastoid [36], and the Lulo cells, which are composed of epithelioid cells and previously described as a possible model for *Leishmania* metabolism and anti-parasitic drug evaluation [37]. Both cells have been reported to be susceptible to *Leishmania* and used as model for vector-parasite interaction, even though the parasite cycle in the insect is extracellular [36–42].

We performed *in vitro* infections of *Wolbachia* using Lulo and LL-5 cell lines, with the aim of obtaining a stable infection. We analysed the expression of immune-related genes upon cell infection. We placed these *Wolbachia* infected cells in contact with *L. infantum* as a first attempt to verify the response against the parasite, which could lead to the possible use of *Wolbachia* against *Leishmania* and a means to control transmission.

## Results

### *Wolbachia* establishment into *L. longipalpis* cell lines

**Fig. 1:**
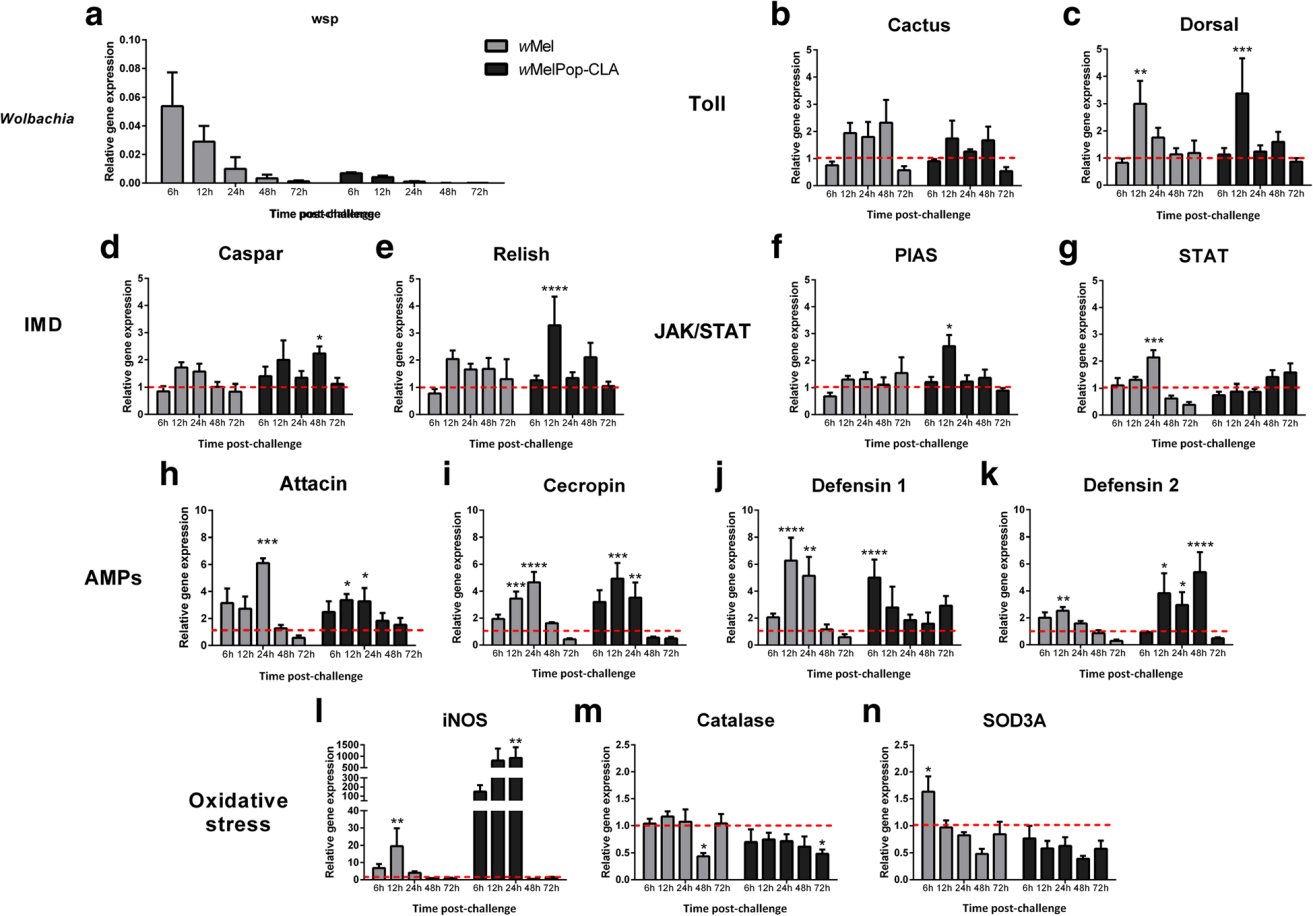
LL-5 sand fly cells immune response after early *Wolbachia* infections (*w*Mel or *w*MelPop-CLA strains). *Wolbachia* detection by wsp relative expression (**a**), Toll pathway regulators Cactus (**b**) and Dorsal (**c**), IMD pathway regulators Caspar (**d**) and Relish (**e**), Jak-Stat regulators (**f** and **g**), AMPs (**h**, **i**, **j** and **k**) and oxidative stress regulation iNOS (**l**), Catalase (**m**) and SOD3A (**n**). Bars represent mean with standard error (SEM) of three biological replicates collected at 6, 12, 24, 48 and 72 h post-early infection. *P < 0.05, **P < 0.01, ***P < 0.001, ****P < 0.0001

The response of *L. longipalpis* LL-5 cells against infection with *Wolbachia* strains *w*Mel and *w*MelPop-CLA was analysed at early stages of interaction for the first 72 h post-infection with the bacteria. The detection of both *Wolbachia* strains decreased gradually from early time-points until 72 h post-infection (Fig. 1a). The expression of immune related genes was evaluated to understand these cells response to early contact with these strains, from 6 to 72 h post-interaction. When compared to the non-infected control group, LL-5 cells responded to *w*Mel increasing the expression of the transcription factors at 12 h post-infection to Dorsal and at 24 h to STAT, while for *w*MelPop, the cells also responded to Dorsal and Relish both at 12 h post-infection (Fig. 1c, e, g). Cactus, which is the repressor of the Toll pathway, did not present a significant expression variation (Fig. 1b), while the expression of Caspar and PIAS, repressors of the IMD and Jak-Stat pathways, increased at 48 and 12 h post-infection, respectively (Fig. 1d, f), in response to wMelPop-CLA infection. For *w*Mel, the AMPs, which are effector molecules of innate immune responses, Attacin at 12 h, Cecropin at 12 h and 24 h, Defensin 1 at 12 h and 24 h and Defensin 2 at 6 h and 12 h, had significantly increased expression (Fig. 1h-k). Post-*w*MelPop-CLA infection, the AMPs increased were Attacin at 12 h and 24 h, Cecropin at 6 h, 12 h and 24 h, Defensin 1 at 6 h and Defensin 2 at 12 h, 24 h and 48 h. In addition, LL-5 cells expressed high levels of iNOS at 12 h post-infection with *w*Mel and at 24 h to *w*MelPop-CLA (Fig. 1l), and low levels of Catalase expression at 48 for *w*Mel and 72 h for *w*MelPop-CLA post-challenges (Fig. 1m). SOD3A expression was not altered after the two *Wolbachia* strains challenges (Fig. 1n), except at 6 h when it was increased post-*w*Mel challenge (see Additional file 1: Table S1 for detailed statistical results).

Later, in order to obtain stable infections of *Wolbachia* in both *L. longipalpis* cells (Lulo and LL-5), experiments were performed initially using only the *w*Mel strain, due to the lower fitness cost caused by this strain in comparison to the *w*MelPop-CLA [32]. In parallel, the mosquito cell line RML-12 was infected with the same *Wolbachia* strain as a control to validate the infection protocol.

**Fig. 2:**
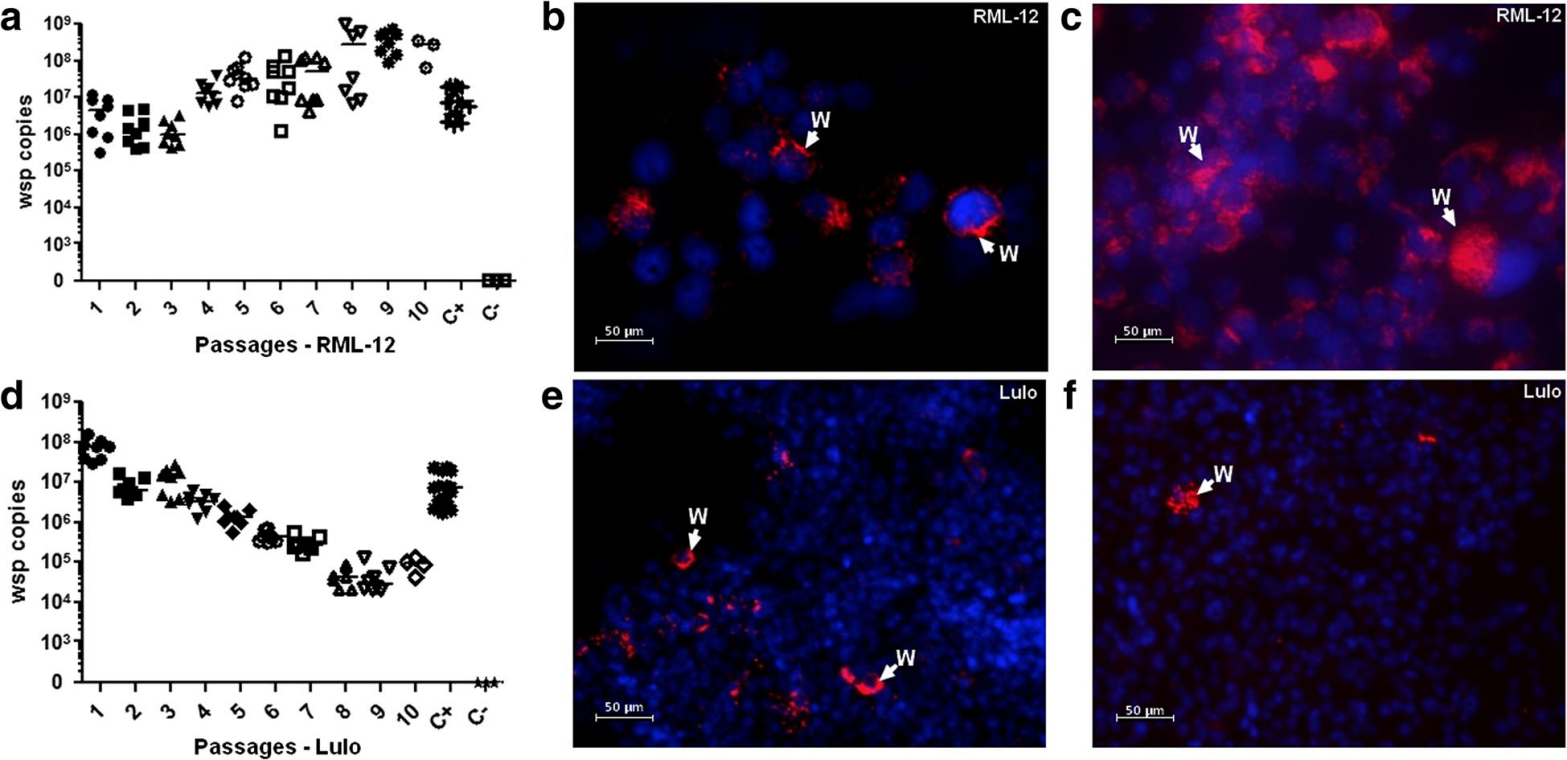
*Wolbachia* infection (*w*Mel strain) into mosquito and sand fly cells. *Wolbachia* introduction into mosquito RML-12 cells showed by absolute quantification (**a**) and by FISH at the 4th passage (**b**) and at 7th passage (**c**) using 40× magnification. In contrast, the decrease of infection into Lulo cells is represented by qPCR (**d**) and by FISH at the 4th passage (**e**), and at the 7th (**f**) in 20× objectives. In **a** and **d**, C+ represents the positive control and C- the negative control. The LL-5 cells showed similar results to Lulo cells (data not shown). The arrows show *Wolbachia*-stained in red and the DNA is stained in blue using DAPI

Numerous attempts to establish the *w*Mel strain in *L. longipalpis* cell lines were unsuccessful, mainly due to high cell mortality after *Wolbachia* infection and slow cell growth. After approximately 62 independent attempts in each cell line, we were able to maintain both cells Lulo and LL-5 in culture after the infection process, and the *Wolbachia* levels were monitored by qPCR in every passage. In subsequent passages, the *w*Mel density gradually decreased, and was only detectable by qPCR in both cell lines up to the 11th passage after transinfection. Once the levels were below detectable limits, the infected cell lines were discarded. Complementing the qPCR results, analysis using FISH to visualise *Wolbachia* confirmed the decrease of *w*Mel over time (Fig. 2d-f).

In contrast, RML-12 cells were able to establish and maintain the *w*Mel infection after a single round of infection. We could detect *w*Mel by qPCR and FISH after the initial infection and could further detect the increase of *Wolbachia* over subsequent cell passages (Fig. 2a-c). These results confirmed the efficiency of the *Wolbachia* extraction protocol and infection, suggesting that the difficulty lies in the combination of *w*Mel and *L. longipalpis* cells rather than the infection protocol or the quality of the *Wolbachia* isolation. Furthermore, the *L. longipalpis* cell lines may be resistant to *w*Mel transinfection. Thereafter, we tried transinfections using the *w*MelPop-CLA strain in both *L. longipalpis* cell lines. It has been previously shown that *w*MelPop-CLA has a higher density in cell lines in comparison to *w*Mel (unpublished data), which could increase the chances of infection into sand fly cells.

**Fig. 3:**
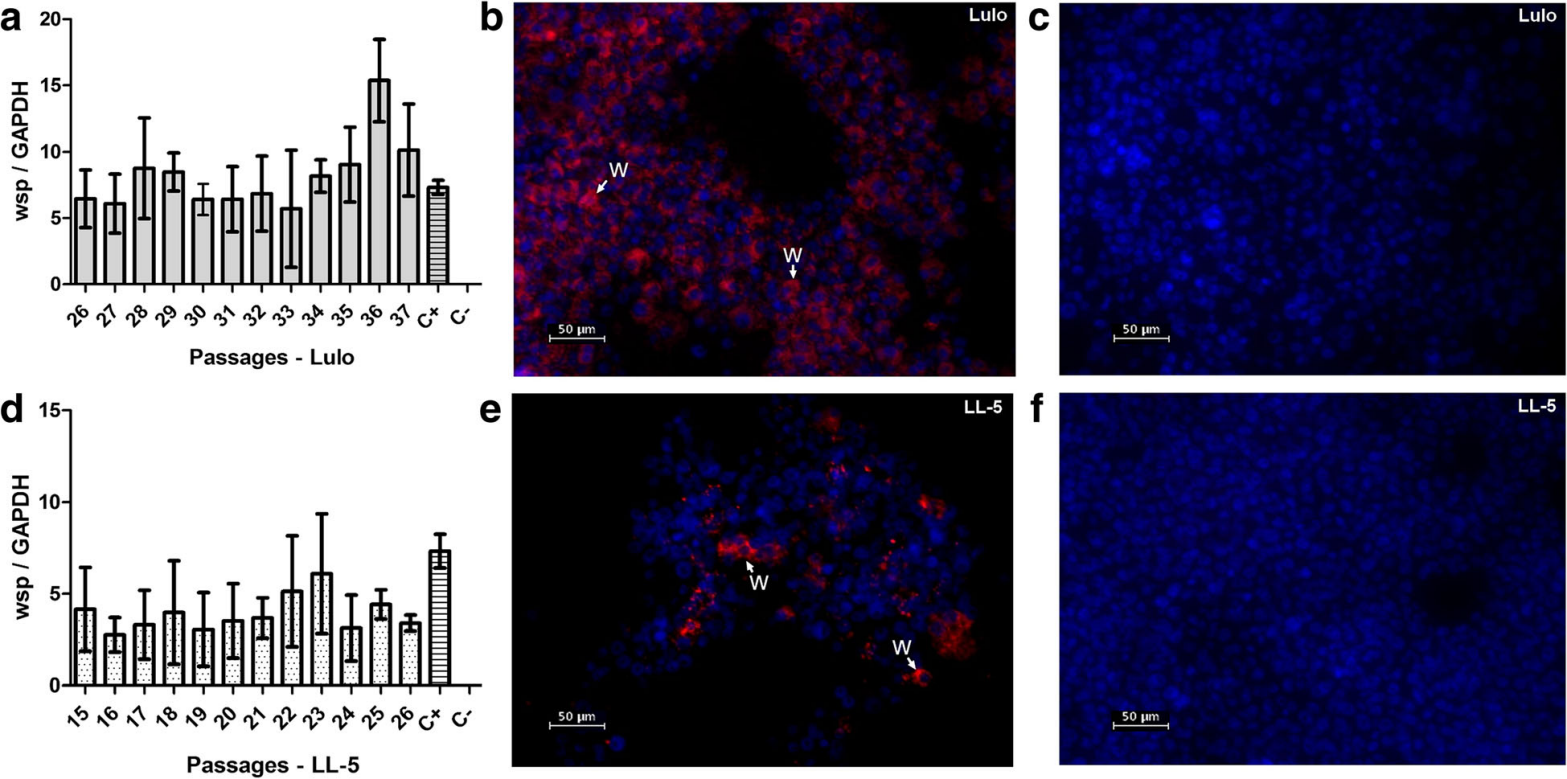
*Wolbachia* establishment (*w*MelPop-CLA strain) into sand fly cells. The bacteria could establish and increase their density, as shown by relative quantification through qPCR (**a** and **d**). In Lulo, the *Wolbachia* density is higher (**a** and **b**), in comparison with LL-5 (**d** and **e**). In (**c**) the Lulo cells control is shown and in **f** the LL-5 control. The cells were observed at 40× magnification, with the arrows pointing at *Wolbachia* (red), whereas the DNA is stained using DAPI (blue)

Around 15 attempts to infect each *L. longipalpis* cell line with *w*MelPop-CLA were performed. In comparison to LL-5 cells, Lulo cells were more susceptible to infection, and after maintaining *Wolbachia* in those cells for over 35 passages, we considered *w*MelPop-CLA successfully established in these sand fly cells. To date, these cells have been maintained for over 70 passages (*c.*1.5 years) with high levels of infection as monitored by relative qPCR and FISH (Fig. 3a, b). By using FISH, it was possible to confirm that the proportion of Lulo-infected cells was very high, around 80%. Although we could see fluctuations of *Wolbachia* density by qPCR among the passages, FISH analysis confirmed that the infection rate remained similar.

After establishment in Lulo, we performed *w*MelPop-CLA infections into LL-5 cells. It was possible to obtain an infection with this strain, as shown in Fig. 3d, e, but it was more difficult to maintain the infection. In those cells, *Wolbachia* densities also showed large fluctuations and, in some cases we lost the infection among the passages. However, the average of *w*MelPop-CLA densities in LL-5 cells were consistently lower in comparison with the same *Wolbachia* strain infections in Lulo cells.

After transinfection with *w*MelPop, we were able to maintain and revive Lulo cells that were frozen and cryogenically stored. After revival of samples which had been frozen for over 6 months, it was possible to re-establish the culture with similar *Wolbachia* densities as they had prior to the freezing process, suggesting that *w*MelPop infections in Lulo cell lines were successfully performed and can be stored for long-term use. However, after thawing aliquots stored in liquid nitrogen, it was difficult of maintain the *Wolbachia* density in similar levels in LL-5 cells.

### *Wolbachia* effect in immune-related gene expression in stable infections of *L. longipalpis* cells

Once stable *Wolbachia* infections in sand fly cells had been obtained, we performed studies to determine whether the introduction of the bacterium could trigger cell immune responses. Once *w*MelPop-CLA was established in both LL-5 and Lulo cell lines, aliquots from each passage were collected for RNA extraction and gene expression analyses. We selected genes from the Toll, IMD and Jak-Stat pathways, and we also evaluated the expression of other immune-related genes including AMPs. For this experiment, all the biological replicates from each cell line had similar *Wolbachia* densities for better comparison among groups, with the average *Wolbachia* density being higher in Lulo than in LL-5 cells (Fig 3a, d). The *w*MelPop-infected LL-5 line showed no significant difference in any of the genes studied compared to their appropriate controls (*P* > 0.05).

**Fig. 4:**
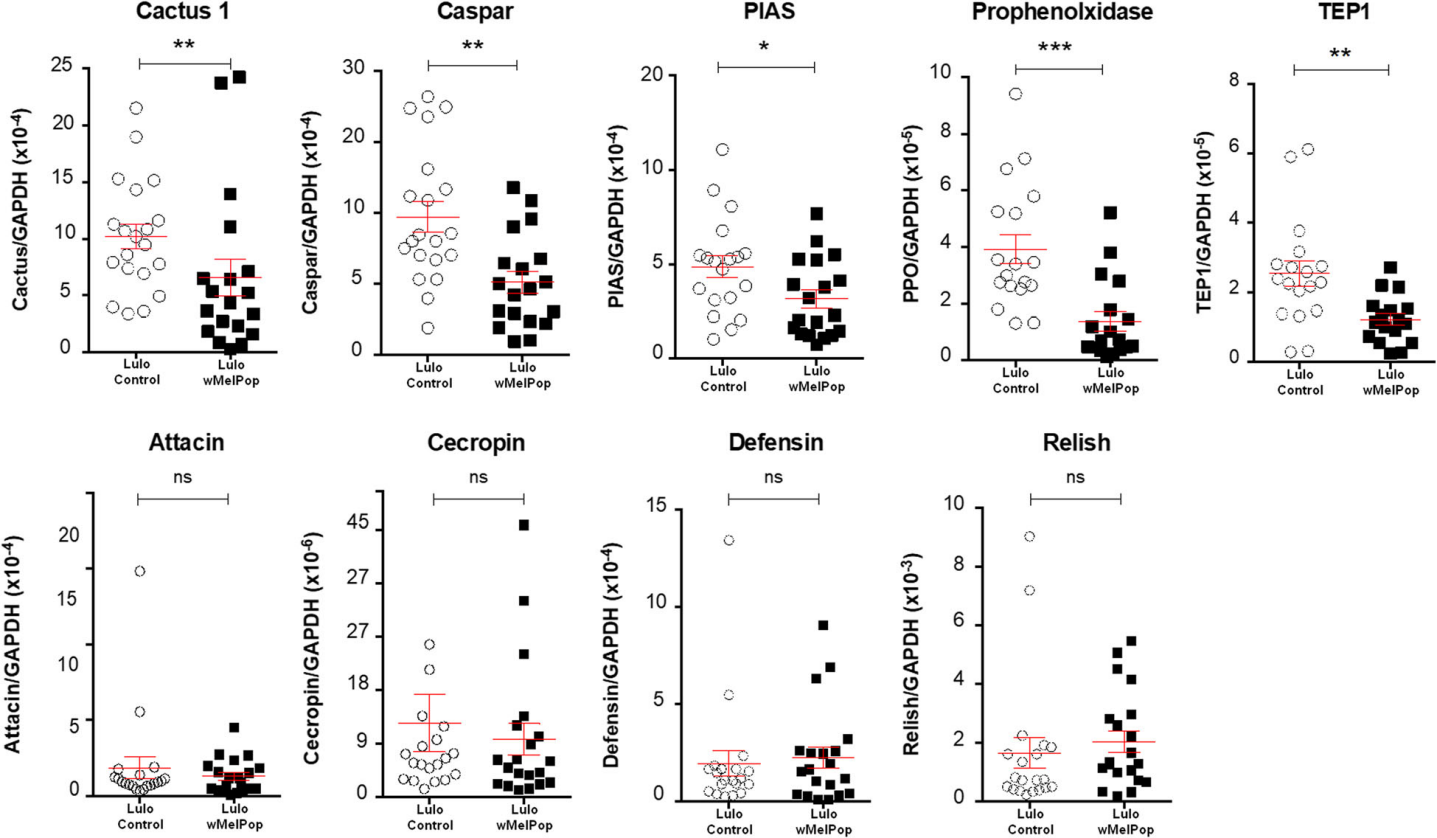
Immune system genes expression in *Wolbachia*-infected Lulo cells after stable infection. Several immune-related genes expression were compared between Lulo cells lines. Lulo cells infected with *w*MelPop-CLA showed significantly lower expression of the genes *Cactus 1*, *Caspar*, *PIAS*, *Prophenoloxidase* and *TEP1.* However, for the gene *Relish*, and the AMPs Attacin, Cecropin and Defensin, there was no difference between both groups (*P* > 0.05). Data were analysed by Mann-Whitney U-test. *P < 0.05, **P < 0.01, ***P < 0.001

Surprisingly, in Lulo cells which had higher *Wolbachia* density than LL-5 cells, gene expression for *Cactus 1* (Mann-Whitney U-test; *U* = 94, *P* = 0.0073), *Caspar* (Mann-Whitney U-test; *U* = 78.5, *P* = 0.0018), *PIAS* (Mann-Whitney U-test; *U* = 109.5, *P* = 0.0396), *Prophenoloxidase* (Mann-Whitney U-test; *U* = 46, *P* = 0.0003) and *TEP1* (Mann-Whitney U-test; *U* = 58, *P* = 0.0018) was significantly lower in comparison with the uninfected Lulo counterparts (Fig. 4). For the genes studied Myd88 (Mann-Whitney U-test; *P* = 0.8441) and Relish (Mann-Whitney U-test; *P* = 0.1806), including the AMPs Attacin (Mann-Whitney U-test; *P* = 0.8604), Cecropin (Mann-Whitney U-test; *P* = 0.5428) and Defensin (Mann-Whitney U-test; *P* = 0.5979), *w*MelPop-infected Lulo cells showed no significant differences compared to the control Lulo cells (*P* > 0.05) (Fig. 4).

### *Leishmania* (*L.*) *infantum* interaction in *Wolbachia*-infected *L. longipalpis* cells

**Fig. 5:**
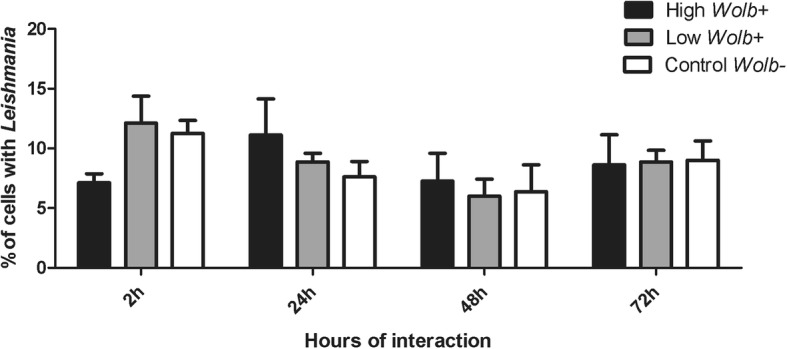
*Leishmania* (*L.*) *infantum* interaction with Lulo cell lines. *Leishmania* interaction with the sand fly cells at 2, 24, 48 and 72 h of co-incubation. The *Leishmania* interaction was performed in three different groups. In black, Lulo cells containing high *Wolbachia* density (ratio 10:1 *Wolbachia* per cell); in grey, Lulo with containing low *Wolbachia* density (ratio 5:1 *Wolbachia* per cell); in white, control Lulo cells (uninfected with *Wolbachia*). Data are expressed in percentile values (%) and represent the average and standard deviation of four independent experiments. Data were analysed by Mann-Whitney U-test and no significant differences were observed between the groups (*P* > 0.05)

Experiments of *Leishmania* interactions into *Wolbachia*-infected cells were performed with high density (10:1 *Wolbachia*:cell) and low density (5:1 *Wolbachia*:cell) *Wolbachia* infections, in comparison to uninfected Lulo cells (controls). We tested parasite load rates of 10:1, 5:1, 1:1 and 0.1:1 parasites per cell. Forty-eight hours post-incubation with *Leishmania*, a large number of cells detached from the glass coverslip at the higher concentrations of parasites (10:1 and 5:1), making it difficult to analyse the results. Due to the loss of cells after exposure to *Leishmania*, we determined that the best concentration for *Leishmania* interaction was the 1:1 ratio (parasite:cell). At two hours of interaction, the cells with high *Wolbachia* density (ratio 10:1) had fewer parasites adhered to the cells in comparison with the other two groups, although with no significant difference (two-way ANOVA; *F*_(6,27)_ = 1.04, *P* = 0.4232). At the same time, the groups with low *Wolbachia* density (ratio 5:1) and the control reached the highest percentage of cells with adhered parasite, in comparison with other time-points, but they were not statistically different (two-way ANOVA; *F*_(2,9)_ = 0.07, *P* = 0.9335). At 24, 48 and 72 h post-incubation, all three groups showed a similar percentage of cells with attached *L. infantum* (two-way ANOVA; *F*_(3,27)_ = 2.10, *P* = 0.1233) (Fig. 5).

## Discussion

*Wolbachia* establishment in cell lines is the first step towards this endosymbiont establishment in novel hosts. This eliminates the need to rear insects through several generations, especially because sand fly rearing is a laborious process and very few productive laboratory colonies are available worldwide. Here we show the first establishment of *Wolbachia* in phlebotomine cell lines. In this study, the introduction and establishment of the bacteria into sand fly cells was difficult, perhaps because the natural host, the brachyceran fly *Drosophila melanogaster*, is not closely related to the nematoceran *L. longipalpis* [43]. Furthermore, it was proposed in previous studies that not only the genetic background, but also the cytoplasmic components of the *Wolbachia-*free cell line are important factors for *Wolbachia* establishment [44].

The first trials to infect LL-5 cells with *w*Mel or *w*MelPop-CLA were not successful and our results indicate that early increased expression of AMPs and oxidative stress may be involved in reducing *Wolbachia* survival within these cells. Previous studies showed that LL-5 cells presented increased immune responses after challenges with heat-killed bacteria and yeast [40]. Together these results indicate that LL-5 cells immune response can have a significant impact on bacterial survival prior to reaching a stable infection.

Initial attempts to obtain a stable *Wolbachia* infection using the strain *w*Mel in sand fly cells failed, probably due to the low density of this strain in mosquito cell lines. In contrast, we could introduce and maintain the strain *w*MelPop in Lulo and LL-5 cell lines, although the density was different in each line*.* The *Wolbachia* infection was maintained at a lower density in LL-5 cells compared to Lulo cells, suggesting Lulo cells are a better model for *in vitro* studies involving *Wolbachia.*

Once established, experiments to determine the expression of immune genes in the presence of *Wolbachia* were performed in both Lulo and LL-5 sand fly cells, which are important defence mechanism of insects against pathogens. Due to a small number of studies involving the immune system activation in sand flies, genes from the innate immune system pathways were selected based on the annotated genome of *L. longipalpis* (https://www.vectorbase.org/organisms/lutzomyia-longipalpis). The genes studied include Cactus and Caspar, which negatively control the Toll and IMD pathways in insects [45, 46] and the gene PIAS which negatively controls the Jak-Stat pathway [47]. Additionally, we studied the prophenoloxidase genes, involved in pathogen melanisation, and TEP1, which codes for a complement-like protein similar to the vertebrate C3b involved in pathogen opsonisation [48].

For stable infections of LL-5 cells, we found that *Wolbachia* had no effect on the expression levels of any of the genes tested in comparison with the uninfected cells, whereas for Lulo, which acquired higher *Wolbachia* density, the presence of the endosymbiont decreased the expression of some genes from the main immune system pathways, such as *Cactus*, *Caspar*, *PIAS*, *PPO* and *TEP1*. Caspar downregulation suggests that upon *Wolbachia* transinfection, this immune pathway may be activated in Lulo cells. After *w*MelPop-CLA introduction, both genes *Cactus* and *PIAS* were downregulated in infected cells in comparison to their respective controls, also suggesting Toll and Jak-Stat activation upon *Wolbachia* infection. These results were the first indication that *Wolbachia* can affect the sand fly immune system pathways in different cascades.

It was previously shown that a reduction of *Caspar* gene expression contributes to the protection of *L. longipalpis* against *Leishmania* infections *in vivo* [49]. In mosquitoes, after knocking down the same gene, infections with *Plasmodium* were decreased [50]. In the present study, *Wolbachia* in Lulo cells significantly reduced the expression of *Caspar*; however, the *Leishmania* load in cells with and without the bacteria remains similar in *in vitro* infection experiments*.*

*In vivo* studies have shown a high antiparasitic activity of the antimicrobial peptide Defensin against *Leishmania* in its natural host *Phlebotomus duboscqi* [51]*.* Similarly, Defensin and Cecropin, other antimicrobial peptides, have the same antiparasitic activity in different hosts infected with a range of parasites [52–54]. For both Lulo and LL-5, there was no difference between the levels of AMPs expressed from *Wolbachia* stably infected and control cells. This same result was previously observed in *Drosophila* and mosquitoes, suggesting that the protection provided by *Wolbachia* is not only based on upregulation of immune system genes from the main pathways and AMPs [55–57].

Experiments with *L. infantum* were performed to test whether the presence of *Wolbachia* in sand fly cells could confer some protection and decrease the number of adhered parasites. Previous studies have shown that Lulo cells are a good model to study *Leishmania* interaction and the parasite life-cycle [41, 43]. As mentioned in previous studies, the nectomonad promastigotes act to establish infection in sand flies by attaching to the midgut wall and then by migrating to the anterior midgut [58, 59]. In 2003, Gossage et al. [60] showed that, in *in vitro* assays, it is possible to obtain the different forms of the parasite, such as procyclic, nectomonad, leptomonad and metacyclic promastigoes. In the present study, we were able to confirm the parasite interaction in both cells with and without the presence of *Wolbachia* (*w*MelPop-CLA). The number of Lulo cells with *Leishmania* remains similar in all three groups in different times post-co-interaction. Our results show that *Wolbachia* does not result in a detrimental effect against *L. infantum* adhesion in *in vitro* assays*.*

*Wolbachia* has been shown to inhibit the replication of dengue, Zika and chikungunya viruses in invertebrate hosts [17, 20, 61, 62] and this is the basis for biocontrol approaches to reduce the burden of these diseases (www.worldmosquito.org). The same has been shown for the parasites *Plasmodium* spp., which need to get into the cells for multiplication and continuation of the life-cycle [19, 31]. It has been speculated that the blocking effect can be due to a number of mechanisms, such as competition between the bacteria and pathogen to invade the host cell and for cellular resources and/or the priming of host immune genes [17, 20, 63].

In contrast, in the sand fly host, the key for a successful transmission of the parasite to the vertebrate host consists in the adhesion of promastigotes to the midgut epithelium using membrane molecules, such as lipophosphoglycan (LPG) and glycosaminoglycan (GAG) [39, 64–66]. In our results, the presence of *Wolbachia* in sand fly cells did not affect the number of *Leishmania* attached to Lulo cells. This was likely due to the lack of competition between *Wolbachia* and *Leishmania* to invade the host cells and for cellular resources, including the lack of upregulation of some immune system from the *L. longipalpis* cell lines.

The establishment and adaptation of *Wolbachia* into cell lines from *L. longipalpis* could potentially facilitate the generation of stably transinfected sand flies to be challenged with *Leishmania*. *In vivo* experiments involving *Wolbachia* and *Leishmania* are important due to the complexity of this organism and the life-cycle of the parasite. To better understand the use of *Wolbachia* against *Leishmania* infection and its possible antiparasitic effects, further experiments must be done with the sand fly invertebrate host to analyse the possibility of using *Wolbachia* as an additional tool to control leishmaniasis*.*

## Conclusions

In this study, we were able to establish a stable infection of *Wolbachia* into *L. longipalpis* cells (Lulo cell line) and we showed that this model is more permissive to the *w*MelPop-CLA than the *w*Mel *Wolbachia* strain. The presence of the bacterium appears to activate the main innate sand fly immune pathways but it does not appear to affect the parasite load of this specific strain of *L. infantum* attached to the cells, in comparison with uninfected Lulo cells.

## Methods

### Cell lines maintenance

Previously established embryonic cell lines from *L. longipalpis*, LL-5 [53] and Lulo [39] were cultured in 25 cm^2^ flasks containing L15 medium (Leibovitz 1963) enriched with 10% tryptose phosphate broth and supplemented with 10% heat inactivated fetal bovine serum (FBS; Gibco, Scoresby, Australia), penicillin (100 U/ml) and streptomycin (100 μg/ml). Cells were incubated at 28 °C without CO_2_. Confluent cell monolayers from both cell lines were mechanically removed using scrapers and passaged at least once per week. In between the passages, the old medium was discarded and fresh medium was added every 3–4 days to avoid the cells being kept for long periods in acidic medium.

The *w*Mel*-* and *w*MelPop-CLA-infected RML-12, including the uninfected RML-12 cell lines from the mosquito species *Aedes albopictus* [66, 44] were obtained from The World Mosquito Program at Monash University (Melbourne, Australia). Both *Wolbachia* strains used to infect these lines were derived from *Drosophila melanogaster*, established in 2008 by McMeniman et al., and were cultured as previously described [29, 44].

### *Wolbachia* purification and introduction into *L. longipalpis* cell lines

The process of infection of *L. longipalpis* cells with *Wolbachia w*Mel and *w*MelPop-CLA was carried out using a modified *Wolbachia* extraction protocol [67]. Briefly, RML-12 cells infected with *w*Mel and *w*MelPop-CLA were first cultured in six 175 cm^2^ flasks containing 20 ml of medium. Cells were grown up to 90% confluence and 70 ml of medium containing cells (≈ 2.5 × 10^8^ cells) was collected and transferred to Falcon tubes for centrifugation at 1000× *g* for 10 min at 4 °C. Pelleted cells were then resuspended in 10 ml of SPG buffer (218 mM sucrose, 3.8 mM KH_2_PO_4_, 7.2 mM K_2_HPO_4_, 4.9 mM L-glutamate, pH 7.2) and sonicated twice for 10 s at 20–25 V on ice. Homogenates were centrifuged at 1000× *g* for 10 min at 4 °C. The supernatant was first filtered through a 2.7 μm Millex syringe filter and then through a 1.2 μm filter (Millipore, Bedford, MA, USA). The filtrate was centrifuged at 14,000× *g* for 15 min at 4 °C to obtain the *Wolbachia* pellet. The bacteria pellet was resuspended in SPG buffer and laid on a monolayer of 80% confluent, uninfected Lulo, LL-5, or RML-12 cells in a 24-well plate. The plate with cells and *Wolbachia* was sealed with parafilm and centrifuged for 60 min at 1500× *g* to increase the contact between the bacteria and the cells and finally incubated at 26 °C. Three days after infection, cells were transferred into a 25 cm^2^ flask containing 4 ml of fresh medium and passaged as described above. Additionally, multiple infections were tested following the same protocol of *Wolbachia* extraction for both *L. longipalpis* cell lines to boost *Wolbachia* infection rates. After the first infection in a 24-well plate, the cells were transferred to 12-well plate to grow until they were 90% confluent and then they were transferred back to 24-well plates for re-infection with *Wolbachia*. Three independent rounds of infection were performed as an attempt to obtain higher infection levels.

### *Wolbachia* purification and immune gene expression in early stage of infection into LL-5 cell lines

The same protocol of *Wolbachia* extraction was performed for the study of early stage of infections in LL-5 cells with both strains. One day before the *Wolbachia* extraction from RML-12 cells, 200 μl of LL-5 cells (≈ 2 ×10^6^ cells) were seeded in a 24-well plate containing 800 μl of fresh medium. After performing the same protocol described above, 100 μl of the extracted *Wolbachia* (*w*Mel and *w*MelPop-CLA) were added to each well and, for control, the same volume of SPG buffer was added, the plate sealed and centrifuged for 60 min at 1500× *g* to increase the contact between the bacteria and the cells, and finally incubated at 26 °C. After infection, the cells were monitored for 6, 12, 24, 48 and 72 h. At each time-point, the cells attached to the 24-well plate were resuspended, centrifuged at 4000× *g* for 5 min, the pellet resuspended in 50 μl of TRIzol reagent (Invitrogen, Carlsbad, USA) and all samples were kept at -80 °C until further experiments. Those samples were thawed at room temperature and homogenized using a 2 mm glass bead on a Mini-Beadbeater-96 (Biospec, Bartlesville, USA) for 30 s. Total RNA was isolated following the manufacturer’s instructions. For cDNA synthesis, 1 μg of RNA was first treated with *DNAse* I (Invitrogen) and the first strand cDNA synthesis was performed by Superscript Reverse Transcriptase III (Invitrogen), both following the manufacturer’s protocol. This assay was performed in three independent experiments. *Wolbachia* relative quantification was performed by qPCR using *wsp* [68] gene expression relative to *L. longipalpis GAPDH* reference gene (ID: LLOJ001891) with primers listed in Table 1. Expression of immune related genes was also performed by qPCR relative to GAPDH gene. Primers used for immunity gene expression such as Cactus and Dorsal (Toll pathway); Caspar and Relish (IMD pathway); PIAS and STAT (Jak-Stat pathway); Attacin, Cecropin, Defensin 2 [69] and Defensin 1 [69] (AMPs); and reactive oxygen species mediated immunity genes Catalase (ID: LLOJ007605), Superoxide Dismutase (SOD3A) (ID: LLOJ008594) and inducible Nitric Oxide Synthase (iNOS) (ID: LLOJ005465) were obtained from referred publications or listed in Table 1. The reactions were performed on a 7500 Real Time PCR System (Applied Biosystems, Foster City, USA) using Power SYBR Green PCR Master Mix (Applied Biosystems) according to the manufacturer’s standard protocol. Each sample, in duplicate, was analysed through the 2-ΔΔCt method. The relative gene expression was expressed as fold change calculated relative to uninfected LL-5 control group. Two-way ANOVA test was used to verify significant differences of relative gene expression in relation to *Wolbachia* infection and time post-infection.

**Table 1:** Primers used in LL-5 cells early infections with wMel and wMelPop-CLA strains

Primer name	Primer sequence (5'-3')	Reference sequence ID
LLiNOS-F	TGGCTGTCGCAATTTGTGTG	LLOJ005465 (VectorBase)
LLiNOS-R	CCGCAATGTTCACCTCAACC	
LLCatalase-F	CGACCGTGGTATCCCTGATG	LLOJ007605 (VectorBase)
LLCatalase-R	AGAAGGCCTCCCCTTTGTTG	
LLSOD3A-F	CCGATAGCGCTGTGAGACAC	LLOJ008594 (VectorBase)
LLSOD3A-R	ATCGGAAATTGCGACCTTGC	
GAPDH-F	TTCGCAGAAGACAGTGATGG	LLOJ001891 (VectorBase)
GAPDH-R	CCCTTCATCGGTCTGGACTA	
wsp-F	TGGTCCAATAAGTGATGAAGAAAC	AF020070.1 (GenBank)
wsp-R	AAAAATTAAACGCTACTCCA	

### *Wolbachia* detection and quantification through PCR amplification in stable infections

On every passage, 200 μl of cells and media were harvested from the flasks to confirm and estimate *Wolbachia* infection rates. Briefly, the cells were centrifuged at 4000× *g* for 5 min, and the pellet resuspended in 50 μl of extraction buffer containing 4 mM EDTA, 20 mM Tris base, 0.4 mM NaCl and 0.25 μg/ml Proteinase K (Bioline, Eveleigh, Australia). The cells were homogenised using a 2 mm glass bead and Mini-Beadbeater-96 (Biospec) for 30 s, and the lysate then incubated at 56 °C for 5 min, followed by a second incubation at 98 °C for 5 min for DNA extraction. Relative quantitative PCR was performed using the primers wspTM_F (5'-CAT TGG TGT TGG TGT TGG TG-3') and wspTM_R (5'-ACA CCA GCT TTT ACT TGA CCA G-3') [70] for *Wolbachia* and GAPDH_F (5'-TTC GCA GAA GAC AGT GAT GG-3') and GAPDH_R (5'-CCC TTC ATC GGT CTG GAC TA-3') for *L. longipalpis*. The reactions were performed on a LightCycler 480 SYBR Green I Master (Roche, North Ryde, Australia) at 95 °C for 5 min, followed by 45 cycles at 95 °C for 30 s, 60 °C for 30 s and 72 °C for 2 s, with an extra 72 °C for 5 min. Each sample, in duplicate, was analysed through the 2-ΔΔCt method by the LightCycler 480 software (Roche).

### *Wolbachia* visualisation by fluorescence *in situ* hybridization (FISH)

Every 3rd or 4th passage after *Wolbachia* infection, 100 μl of the sand fly cells at 90% confluence was transferred to an 8-well chamber slide (Thermo Fisher, Riverstone, Australia) containing 400 μl of media and incubated at 26 °C for at least 3 h, to allow cell adhesion prior to the FISH assay. After incubation, cells were processed by FISH as previously described [44]. Briefly, cells were fixed for 10 min in freshly prepared 4% formaldehyde in 1× PBS buffer with 0.5 % Triton X-100, washed 3 times in 1× PBS for 5 min each and incubated in absolute ethanol for 5 min. This was followed by the hybridization process conducted overnight at 37 °C with a hybridization buffer [50% formamide, 5× saline-sodium citrate (SSC), 200 g/l dextran sulfate, 250 mg/l poly(A), 250 mg/l salmon sperm DNA, 250 mg/l tRNA, 0.1 M of DTT (1,4-dithiothreitol), 0.5× Denhartdt’s solution] containing 200 ng of each of the specific *Wolbachia* probes for *16S* rRNA (W2: 5'-CTT CTG TGA GTA CCG TCA TTA TC-3' and W3: 5'-AAC CGA CCC TAT CCC TTC GAA TA-3'), labelled by Rhodamine at the 5' end [71]. After hybridization, samples were washed twice in 10 mM DTT in 1× SSC and then twice in 10 mM DTT in 0.5× SSC at 55 °C and for 15 min each, followed by a wash with 10 mM DTT in 0.5× SSC, at room temperature. To observe DNA, cells were washed in 10 mM DTT in 0.5× SSC supplemented with 10 mg/ml DAPI (4,6- diamidino-2-phenylindole, dihydrochloride) for 10 min at room temperature, rinsed three times in Mili-Q water, and mounted on a glass slide with Prolong Gold (Life Technologies, Scoresby, Australia). Samples were viewed under an epifluorescence microscope (AXIO Imager II, Zeiss, Le Pecq, France) equipped with Axiocam, using 20× and 40× objectives.

### Immune gene expression in stable infections in sand fly cells

From passage 18th onwards (approximately 18 weeks after infection), in each passage 200 μl of cells and media at 90% confluence were harvested (≈ 4 × 10^6^ cells), centrifuged at 4000× *g* for 5 min, the pellet resuspended in TRIzol reagent (Invitrogen) and all samples kept at -80 °C until further experiments. Samples with similar *Wolbachia* density were selected for immune system gene expression experiments. Those samples were thawed at room temperature and homogenized using a 2 mm glass bead on a Mini-Beadbeater-96 (Biospec) for 30 s. Total RNA was isolated following the manufacturer’s instructions. For cDNA synthesis, 2 μg of RNA was first treated with DNAse I (Invitrogen) and the first strand cDNA synthesis was performed by the Superscript Reverse Transcriptase III (Invitrogen), both following the manufacturer’s protocol. For immune system expression, primers for genes from different immune pathways were designed using the Primer-BLAST tool (NCBI, https://www.ncbi.nlm.nih.gov/tools/primer-blast/). The sequences were based on the *L. longipalpis* annotated genome available at VectorBase. Gene IDs used for primer design are as follows: Cactus1 (ID: LLOJ004612), Caspar (ID: LLOJ002950), PIAS (ID: LLOJ002593-RA), Prophenoloxidase (ID: LLOJ001742) and TEP1 (ID: LLOJ007923). Furthermore, primers designed for the genes *Relish*, the antimicrobial peptide genes (AMPs) *Attacin*, *Cecropin* and *Defensin* previously described [40] were also included in this study. The same conditions for quantitative PCR were applied, as previously described in this study for *Wolbachia* density, and each sample was performed in duplicate. Expression analysis was performed through the relative quantification using qGENE and normalized to GAPDH. Statistical significance between all data sets was determined using the Mann-Whitney U-test (Graph Pad Prism, version 5.03).

### *Leishmania* interaction with Lulo cells

To test the effect of *Wolbachia* on *Leishmania* adhesion, we used Lulo cells with and without *Wolbachia*, and promastigotes of *L. infantum* (MHOM/BR/1974/PP75). We tested two different densities of *Wolbachia-*infected cells to investigate whether the amount of bacteria in those cells could interfere with the parasite adhesion and interaction with the sand fly cell lines. The parasites were grown in Schneider’s media (Gibco) supplemented with 10% v/v heat-inactivated FBS (Gibco), 1% v/v GlutaMAX (Gibco), 1% v/v BME Vitamins solution 100× (Sigma-Aldrich, Castle Hill, Australia), 2% sterile male urine and penicillin (100 U/ml) and streptomycin (100 μg/ml) (Gibco), maintained at 28 °C without CO_2_. The assay was performed as previously described with some modifications [43, 44]. Briefly, Lulo cells infected and uninfected with *Wolbachia* were seeded on glass coverslips in a 24-well plate, to a final number of 2 × 10^6^ cells per well, one day before the interaction with parasites. For the cell/promastigote interaction assay, different concentrations of *L. infantum* and cells were tested (10:1, 5:1, 1:1 and 0.1:1) for standardisation and a ratio of about 1:1 parasite/cell was used. After 2 h of co-incubation and interaction, the non-adhered promastigotes were washed off with phosphate buffered saline (PBS) pH 7.2 and the cells were monitored for 2, 24, 48 and 72 h. At each time-point, the cells attached to the coverslip were fixed with methanol and stained with Quick Dip Field Staining (Thermo Fisher, Riverstone, Australia) and mounted with Canada Balsam (Sigma-Aldrich) in slides for further analysis. Three independent experiments were performed.

After conducting all the time-point collections, the number of promastigotes attached per cell in both *Wolbachia-*infected and uninfected lines was determined by counting 200 cells per coverslip under a light microscope (AXIO Imager II, Zeiss) equipped with Axiocam, using 100× objectives. This assay was performed in four independent experiments and the results were expressed as the percentage of cells with the parasite at different times post-exposure to the parasite. The statistical analysis was performed using two-way ANOVA test to verify significant differences of the *L. infantum* adhesion in relation to *Wolbachia* infection and time post-infection, and also Bonferroni *post-hoc* tests, both using GraphPRISM software (version 5.03).

## Additional file

Additional file 1: **Table S1.** Statistical analysis of LL-5 sand fly cells immune response after early *Wolbachia* infections (*w*Mel or *w*MelPop-CLA strains). (DOCX 20 kb)

## Abbreviations

AMPs: Antimicrobial peptides
cDNA: Complementary deoxyribonucleic acid
CHIKV: Chikungunya virus
DENV: Dengue virus
DNA: Deoxyribonucleic acid
FISH: Fluorescence *in situ* hybridization
GAG: Glycosaminoglycan
LPG: Lipophosphoglycan
PBS: Phosphate-buffered saline
qPCR: Quantitative polymerase chain reaction
RNA: Ribonucleic acid
rRNA: Ribosomal ribonucleic acid
VL: Visceral leishmaniasis
