# Supplementary material for: Wolbachia introduction into Lutzomyia longipalpis (Diptera: Psychodidae) cell lines and its effects on immune-related gene expression and interaction with Leishmania infantum
Source: Parasit Vectors. 2019 Jan 15;12:33. doi: 10.1186/s13071-018-3227-4 (PMC6332621; doi:10.1186/s13071-018-3227-4)
Supplement: Supplementary file 1 — Table S1. Statistical analysis of LL-5 sand fly cells immune response after early Wolbachia infections (wMel or wMelPop-CLA strains). (DOCX 20 kb) [file 13071_2018_3227_MOESM1_ESM.docx]

**Additional file 1: Table S1.** Statistical analysis of LL-5 sand fly cells immune response after early *Wolbachia* infections (*w*Mel or *w*MelPop-CLA strains).

| **Figure** | ***Wolbachia* strain** | **LL-5 Gene** | **Time post-infection** | **Column factor *F*_(df1,df2)_** | **Row factor *F*_(df1,df2)_** | ***P*-value** |
| --- | --- | --- | --- | --- | --- | --- |

| Fig. 1 c | wMel | Dorsal | 12h | *F*_(1, 46)_ = 3.310 | *F*_(4, 46)_ = 2.535 | 0.0032 |
| --- | --- | --- | --- | --- | --- | --- |
| Fig. 1 c | wMelPop-CLA | Dorsal | 12h | *F*_(1, 68)_ = 7.363 | *F*_(4, 68)_ = 4.718 | 0.0002 |
| Fig. 1 d | wMelPop-CLA | Caspar | 48h | *F*_(1, 68)_ = 14.13 | *F*_(4, 68)_ = 1.999 | 0.0123 |
| Fig. 1 e | wMelPop-CLA | Relish | 12h | *F*_(1, 68)_ = 16.56 | *F*_(4, 68)_ = 3.847 | < 0.0001 |
| Fig. 1 f | wMelPop-CLA | PIAS | 12h | *F*_(1, 68)_ = 6.315 | *F*_(4, 68)_ = 1.542 | 0.0258 |
| Fig. 1 g | wMel | STAT | 24h | *F*_(1, 39)_ = 1.474 | *F*_(4, 39)_ = 6.716 | 0.0003 |
| Fig. 1 h | wMel | Attacin | 24h | *F*_(1, 38)_ = 13.49 | *F*_(4, 38)_ = 4.716 | 0.0001 |
| Fig. 1 h | wMelPop-CLA | Attacin | 12h | *F*_(1, 65)_ = 18.56 | *F*_(4, 65)_ = 1.416 | 0.0205 |
| Fig. 1 h | wMelPop-CLA | Attacin | 24h | *F*_(1, 65)_ = 18.56 | *F*_(4, 65)_ = 1.416 | 0.0263 |
| Fig. 1 i | wMel | Cecropin | 12h | *F*_(1, 44)_ = 25.59 | *F*_(4, 44)_ = 7.524 | 0.0006 |
| Fig. 1 i | wMel | Cecropin | 24h | *F*_(1, 44)_ = 25.59 | *F*_(4, 44)_ = 7.524 | < 0.0001 |
| Fig. 1 i | wMelPop-CLA | Cecropin | 6h | *F*_(1, 68)_ = 19.62 | *F*_(4, 68)_ = 4.578 | 0.0208 |
| Fig. 1 i | wMelPop-CLA | Cecropin | 12h | *F*_(1, 68)_ = 19.62 | *F*_(4, 68)_ = 4.578 | 0.0008 |
| Fig. 1 i | wMelPop-CLA | Cecropin | 24h | *F*_(1, 68)_ = 19.62 | *F*_(4, 68)_ = 4.578 | 0.0051 |
| Fig. 1 j | wMel | Defensin 1 | 12h | *F*_(1, 44)_ = 15.00 | *F*_(4, 44)_ = 4.602 | < 0.0001 |
| Fig. 1 j | wMel | Defensin 1 | 24h | *F*_(1, 44)_ = 15.00 | *F*_(4, 44)_ = 4.602 | 0.0029 |
| Fig. 1 j | wMelPop-CLA | Defensin 1 | 6h | *F*_(1, 68)_ = 18.22 | *F*_(4, 68)_ = 5.039 | < 0.0001 |
| Fig. 1 k | wMel | Defensin 2 | 6h | *F*_(1, 45)_ = 10.33 | *F*_(4, 45)_ = 3.538 | 0.036 |
| Fig. 1 k | wMel | Defensin 2 | 12h | *F*_(1, 45)_ = 10.33 | *F*_(4, 45)_ = 3.538 | 0.0069 |
| Fig. 1 k | wMelPop-CLA | Defensin 2 | 12h | *F*_(1, 66)_ = 22.77 | *F*_(4, 66)_ = 6.094 | 0.0205 |
| Fig. 1 k | wMelPop-CLA | Defensin 2 | 24h | *F*_(1, 66)_ = 22.77 | *F*_(4, 66)_ = 6.094 | 0.0237 |
| Fig. 1 k | wMelPop-CLA | Defensin 2 | 48h | *F*_(1, 66)_ = 22.77 | *F*_(4, 66)_ = 6.094 | < 0.0001 |
| Fig. 1 l | wMel | iNOS | 12h | *F*_(1, 42)_ = 4.197 | *F*_(4, 42)_ = 2.008 | 0.0034 |
| Fig. 1 l | wMelPop-CLA | iNOS | 24h | *F*_(1, 65)_ = 5.606 | *F*_(4, 65)_ = 2.494 | 0.001 |
| Fig. 1 m | wMel | Catalase | 48h | *F*_(1, 46)_ = 0.6160 | *F*_(4, 46)_ = 3.003 | 0.0149 |
| Fig. 1 m | wMelPop-CLA | Catalase | 72h | *F*_(1, 50)_ = 17.85 | *F*_(4, 50)_ = 0.2995 | 0.0408 |
| Fig. 1 n | wMel | SOD3A | 6h | *F*_(1, 42)_ = 1.092 | *F*_(4, 42)_ = 4.550 | 0.0128 |
